# Energy Expenditure in Older People Hospitalized for an Acute Episode

**DOI:** 10.3390/nu11122946

**Published:** 2019-12-04

**Authors:** Marc Bonnefoy, Thomas Gilbert, Sylvie Normand, Marc Jauffret, Pascal Roy, Béatrice Morio, Catherine Cornu, Sylvain Roche, Martine Laville

**Affiliations:** 1 Department of Geriatric Medicine, Groupement Hospitalier Sud, CHU de Lyon, 69495 Bénite-Pierre CEDEX, France; thomas.gilbert@chu-lyon.fr (T.G.); mjauffret001@gmail.com (M.J.); 2 CarMeN, U1060 INSERM, 69921 Oullins CEDEX, France; beatrice.morio@lyon.inra.fr (B.M.); martine.laville@chu-lyon.fr (M.L.); 3 Rhône-Alpes Center for Research in Human Nutrition, European Center for Nutrition and Health, Groupement Hospitalier Sud, CHU de Lyon, 69495 Pierre-Bénite CEDEX, France; sylvie.normand@chu-lyon.fr; 4 Université Claude Bernard Lyon 1, 69100 Villeurbanne, France; 5 HESPER, EA 7425 Université Claude Bernard lyon 1, 69373 Lyon 8 CEDEX, France; 6 Department of Biostatistics, Health sciences department, Hospices Civils de Lyon, 69003 Lyon, France; pascal.roy@chu-lyon.fr (P.R.); sylvain.roche02@chu-lyon.fr (S.R.); 7 CNRS UMR 5558, Laboratory of Biometry and evolutive Biology, Biostatistics and health, 69100 Villeurbanne, France; 8 Center of clinical investigations, Hôpital Louis Pradel, 69500 Bron, France; catherine.cornu@chu-lyon.fr; 9 Department of endocrinology and Nutrition, Groupement Hospitalier Sud, CHU de Lyon, 69495 Pierre-Bénite CEDEX, France

**Keywords:** doubly labeled water, energy expenditure, energy requirements, energy metabolism, body composition, aged, frail elderly

## Abstract

Weight loss and worsening of nutritional state is a frequent downfall of acute hospitalization in older people. It is usually accepted that acute inflammation is responsible for hypercatabolism. However, several studies suggest, on the contrary, a reduction in resting energy expenditure (REE). This study aimed to obtain a reliable measure of REE and total energy expenditure (TEE) in older patients hospitalized for an acute episode in order to better assess patients’ energy requirements and help understand the mechanisms of weight loss in this situation. Nineteen hospitalized older patients (mean age 83 years) with C-reactive protein (CRP) level >20mg/L were recruited. REE and TEE were measured using gold standard methods of indirect calorimetry and doubly labeled water (DLW), respectively. REE was then compared to data from a previous study on aged volunteers from nursing homes who were free of an acute stressor event. Energy requirements measured by DLW were confirmed at 1.3 × REE. Energy intake covered the needs but did not prevent weight loss in these patients. TEE was not increased in hospitalized patients and was not influenced by inflammation, while the relationship between REE and inflammation was uncertain. Our results suggest that lean mass remains the major determinant of REE in hospitalized older people and that weight loss may not be explained solely by a state of hypercatabolism.

## 1. Introduction

Age and multiple chronic conditions [1] are major risk factors for unplanned hospitalization after acute illness [2]. Malnutrition can affect more than 40% of hospitalized patients aged 65 years and older [3]. In a recent prospective study, a Charlson comorbidity index over two was an independent predictor of malnutrition at hospital admission [4]. Malnutrition is associated with poor outcomes in hospitalized older patients, leading to increased mortality risk, longer length of stay, and higher costs [3]. A negative balance between food intake and/or increased energy expenditure may be a major contributor of weight loss during an acute condition [5].

Maintaining an appropriate energy balance in this high-risk population depends on a precise knowledge of patients’ energy requirements. Yet, this is not as straightforward as it seems. A wide variety of formulae has been developed to estimate patients’ resting energy expenditure (REE) [6,7]. However, these are mostly based on healthy non hospitalized persons. Furthermore, many studies have shown discrepancies between estimated and measured REE in hospitalized older patients [5,7]. Data on energy requirements of old and/or malnourished acutely ill patients are scarce. More often, studies refer to chronic diseases [8,9,10,11,12].

Acute illnesses may increase energy expenditure because of increased metabolic turnover and hypermetabolic effects of fever or inflammation. However, anorexia-induced energy restriction may conversely decrease REE [13]. According to the current state of knowledge, ageing is accompanied by a reduction in resting and total energy expenditure (TEE) [14], but very scarce data are available with precise measurements of TEE. Little is known about energy requirements and expenditure in acutely ill very old patients; thus, there is a need for studies that would help understand the metabolic response to tissue inflammation in case of acute illness.

The most accurate method for determining energy requirements in hospitalized patients is indirect calorimetry for REE [15] and the doubly labeled water method (DLW) for TEE [16,17], although the latter method is difficult to apply in routine practice. Proxy measures of TEE usually rely on estimated REE and presumed physical activity level (PAL), which leads to two potential levels of approximation. In particular, no equation is sufficiently accurate to predict REE in most hospitalized patients [15,18], and there is only very limited data on PAL in older patients [19].

The primary goal of this work was to measure TEE in acutely ill patients with the DLW method to study very precisely their energy requirements and energy balance. The second objective was to compare indirect calorimetry measures of REE in hospitalized acutely ill older patients versus control, stable older patients living in nursing homes as well as to examine the different contributors to REE and the role of inflammation more specifically.

## 2. Materials and Methods

### 2.1. Participants and Setting

We conducted an observational cross-sectional study in Lyon Sud University Hospital. Nineteen volunteer older patients (age ≥65 years) hospitalized in acute or rehabilitation care unit with an acute condition, malnutrition, and an ongoing inflammatory process were included (study group, hereafter referred to as group A). Inclusion criteria were as follows: C-reactive protein (CRP) ≥20mg/L and duration of hospitalization ≥14 days. Exclusion criteria were as follows: unstable medical condition (e.g., decompensated heart failure, severe renal insufficiency) or patients at end of life, subjects receiving corticosteroid treatment or insulin therapy, and subjects with severe dementia (Mini-Mental State Examination score (MMSE) <15).

Only this group had their TEE measurement done by the doubly labeled water technique. Inclusions ranged from June 2006 to June 2010.

The lack of available data using the DLW method to measure TEE for this population at the time the study was conceived did not allow us to estimate a precise sample for our study.

### 2.2. Control Population

For secondary objectives, group A was compared to data retrieved from a previous study (control group B) [20]. Group B included 55 volunteer patients over 70 years of age residing in nursing homes who did not have a severe chronic disease or acute pathology in progress and were not malnourished. Therefore, it appeared particularly interesting as a control group for the acute hospital stress context encountered in group A. Exclusion criteria were as follows: uncontrolled or rapidly evolving diseases, dementia, type 1 diabetes, severe renal insufficiency (blood creatinine over 200 mol/L), severe functional limitation preventing exercising, long-term corticosteroid therapy, and age under 72 years. Details of the recruitment and methods have been reported previously [20].

### 2.3. Measurements

On day 1 of the study, characteristics and blood parameters of patients were collected, namely, age, sex, weight, height, body mass index (BMI), CRP, orosomucoid, albumin, and prealbumin. Scores obtained from the Mini Nutritional Assessment (MNA) were reported [21,22]. Food intake and CRP on day 14 were only measured in group A. The same certified dietitian calculated mean total daily calorie intake (protein, fat, carbohydrate) over two three-day periods from day 1 to day 3 and again from day 11 to day 14 using French food composition tables (BILNUT 4.0, S.C.D.A. Nutrisoft 1995, Cerelles, France). Characteristics (at inclusion of the previous study [20]) collected in the control population (group B) were age, weight, height, BMI, REE by indirect calorimetry, body composition from total body water (TBW) measured by H_2_^18^O dilution, energy intake, and MNA. REE and TBW were measured by the same laboratory using methods reported above. Given the high costs of the DLW method, TEE was not measured in this group.

For both groups, body composition was calculated from TBW measurement after isotopic dilution. In group A, postdose plasma samples were collected 3 and 4 h after doubly labeled water ingestion (^2^H_2_^18^O) for the determination of TBW using the isotopic dilution method. TBW was calculated from the average of the dilution spaces of deuterium (^2^H) and oxygen-18 (^18^O) after correction for isotope exchange by 1.041 and 1.007, respectively [23]. Similarly, for group B, a H_2_^18^O (2.5% ^18^O) dilution was used, as described previously [20].

In both groups, the fat-free mass (FFM) compartment was calculated assuming a hydration level of 73.2%. Fat mass was obtained from the difference between body weight (BW) and FFM and was expressed as a percentage of body mass.

All patients had their REE measured. It was measured in the morning of day 1 by indirect calorimetry (Deltratac Monitor MBM-100; Datex Instrumentary Corporation, Helsinki, Finland) with an open-circuit indirect calorimeter using the ventilated hood method for one hour after 30 min adaptation. This method relies on the measurement of oxygen consumption and carbon dioxide production. Indirect calorimetry was performed in patients in supine position at complete rest after a 12 h overnight fast. Machine calibration was performed against test gas of 96% oxygen and 4% carbon dioxide. Steady state was obtained when the variation coefficient for oxygen consumption and carbon dioxide production measures was 5% or less across five consecutive minutes. Urine samples were obtained before this test to determine N excretion by chemoluminescence (Antek 703C; Sopares, Paris, France) [24]. REE was calculated from indirect calorimetry principles using Ferrannini’s equations [25]. In addition, an estimation of REE was calculated in patients from group A of hospitalized patients using Harris and Benedict’s formula [26] in order to compare estimated and measured REE.

In group A, TEE was measured using the gold standard DLW method [17]. The DLW technique uses a mixture of water labeled with two stable isotopes: deuterium and oxygen-18 (Eurisotop, St Aubin, France) [27]. On the morning of day 1, after baseline plasma sample collection to determine baseline isotope enrichment, a premixed dose of 0.07 g/kg BW ^2^H_2_O (99.9% 2H) and 1.5 g/kg BW H_2_^18^O (10% ^18^O) was administered to the subjects followed by a water rinse. Postdose plasma was sampled at 3 and 4 h time points for determination of TBW from isotope dilution space. Then, on the mornings of days 8 and 15, enriched plasma samples were collected to determine elimination rates for the two isotopes (^2^H and ^18^O). All samples were stored at −20 °C in cryogenically stable tubes until analysis by isotope ratio mass spectrometry. Five milliliter blood samples were collected and then centrifuged (4 °C, 15 min at 3500 rpm) for plasma separation. Water from plasma samples was then extracted by centrifugation (4 °C, 35 min at 3500 rpm) on a polyesthersulfone (PSE) membrane (Vivaspin 2 mL Concentrator, Sartorius, Germany). Continuous flow equilibration analyses were performed using a multiflow system connected to an Isoprime isotope ratio mass spectrometer (IRMS) (Isoprime Ltd., Cheadle, UK). Samples were prepared in triplicate for ^2^H analyses and in duplicate for ^18^O analyses and were each injected three times.

TEE was determined using the two-point method according to Schoeller et al. [17]. After determining TBW from average dilution spaces of ^2^H and ^18^O [23], the production of CO_2_ was calculated according to the equation of Schoeller et al. [17]. TEE was then derived using Weir’s equation [28] with fasting respiratory quotient (RQ) measured by indirect calorimetry.

Finally, PAL was estimated by calculating TEE/REE.

### 2.4. Statistical Analysis

Each subject characteristic was summarized by its mean and standard deviation (quantitative variable) or frequency and percentage (ordinal variable). Measured TEE and mean energy intake were compared using Wilcoxon’s test on matched data. Correlations between quantitative variables were assessed using Spearman’s correlation coefficient because some variables, such as CRP, showed asymmetrical distributions. The relationship between REE and FFM, CRP, age, and sex was assessed by a multiple linear regression model. To ease interpretation, the effect of age was expressed per five-year increment, the effect of FFM was expressed per 5 kg increment, and the effect of CRP was expressed per 20 unit increment. After adjustment for all variables, the regression coefficients relative to age, FFM, and CRP may be respectively interpreted as differences in REE per variable increment.

To further assess the associations between inflammation and energy expenditure, the difference in CRP levels between day 14 and day 1 (CRPdiff) was calculated for each of the 19 patients in group A, and partial Spearman’s correlation coefficients between CRPdiff, REE, and TEE were calculated, adjusted for FFM, age, and sex.

All analyses used Stata software, version 13 (Stata Corp, College Station, TX, USA). All tests were bilateral, and *p* < 0.05 was considered for statistical significance.

### 2.5. Ethics

The protocol of the study was approved by the ethics committee (LYON B 2002-112B, approved 10 February 2003). Written informed consent was obtained for each patient.

## 3. Results

### 3.1. Patient Characteristics

Group A (*n* = 19) comprised 13 women and six men who were hospitalized for various usual reasons (e.g., fall, chest infection, or urinary tract infection). Patients’ characteristics are presented in Table 1. They were malnourished with a mean MNA score of 18.2 (standard deviation (SD) = 3.8) and mean albumin levels of 29 g/L (SD = 5). They had raised inflammatory markers, with mean CRP levels of 62 mg/L (SD = 32) and mean orosomucoid of 1.71 g/L (SD = 0.63). Out of the 55 patients in group B, 48 were women. These patients did not show any malnutrition criteria; the mean value for BMI was 27.0 kg/m^2^ (SD = 4.3), mean MNA score was 24.6 (SD = 1), and the lowest albumin value was 37 g/L (mean albumin 41 g/L; SD = 2). With regard to body composition, both groups were generally comparable, although fat mass was slightly lower (2 kg) in group A. Further details on anthropometric characteristics according to sex are given in Table 2.

### 3.2. Energy Balance and Physical Activity Level

Table 3 presents the measured energy intake and expenditure for patients in group A. Hospitalized patients had a mean weight loss of 1.3 kg (SD = 1.8) within 14 days of follow-up. However, mean energy intake levels were stable between the first three days and last three days, that is, 23 kcal/kg BW/d (*p* = 0.42). In group A, energy intake was comparable to the measured TEE (Wilcoxon test *p* = 0.39).

REE values were similar in both groups. On average, REE was 176.60 kcal/day higher in women than in men, but this difference was not found to be statistically significant (*p* = 0.78; data not shown). There was poor agreement between REE either measured by indirect calorimetry or estimated by Harris and Benedict formula [26] (see Appendix A).

Mean TEE was measured at 1497 kcal/d, that is, 24 kcal/kg BW/d and 39 kcal/ kg FFM/d.

PAL in group A of hospitalized patients with acute inflammation was calculated at 1.3.

### 3.3. Associations between REE, Fat-Free Mass, and CRP

For example, a man aged 83 years with a FFM equal to 38.1 kg and a CRP equal to 0.5 mg/L had a REE equal to 1108 kcal/day (Table 4). Also, on average, REE increased by 20 kcal/day per 20 mg/L increase in CRP. However, this association was not found to be statistically significant (*p* = 0.086).

In contrast, on average, the REE increased by 103 kcal/day per 5 kg increase in FFM and decreased by 31 kcal/day per five-year increase in age (*p* < 0.001 and *p* = 0.03, respectively).

Appendix B shows the results of the bivariate analyses prior to the modelization. There was a good correlation between FFM and REE, but correlation was low between CRP levels and REE (Spearman’s correlation coefficient 0.71 and 0.17, respectively).

Among the 19 patients in group A, the mean difference between the CRP level at day 14 and on admission (CRPdiff) was −39.16 (SD = 45.31). The nonparametric rank correlation study showed a significant negative correlation between CRPdiff and REE (Spearman’s coefficient *ρ* = −0.51; *p* = 0.03). In other words, as the CRPdiff values were almost all negative, the more the CRP at day 14 tended to decrease compared to that at baseline, the higher the baseline REE tended to be; conversely, the less the CRP at day 14 tended to decrease compared to that at baseline, the lower the baseline REE tended to be. In contrast, no significant association was found between CRPdiff and TEE (*ρ* = −0.05; *p* = 0.84).

Adjustment for FFM, age, and sex did not change the results: Spearman’s partial correlation between CRPdiff and REE was significant (*ρ* = −0.51; *p* = 0.05), while Spearman’s partial correlation between CRPdiff and TEE was not significant (*ρ* = 0.10; *p* = 0.72).

## 4. Discussion

For the first time, to the best of our knowledge, in older patients hospitalized for acute or subacute event with inflammatory process, PAL was determined with the gold standard DLW method as 1.3 ± 0.2, with daily energy intake (1420 kcal/d) being just sufficient to cover the daily energy requirements (1497 kcal/d). Our data did not show that TEE was increased in acutely or subacutely ill old patients with inflammatory process and malnutrition. However, although we observed relatively low values of REE, a negative association was found between the evolution of CRP and REE at baseline. These results could suggest a possible influence of inflammation on REE, although this could not definitely be confirmed.

REE declines with age in older adults, as many studies have shown [29,30,31]. Generally, this decline is partly the result of the decrease in FFM, and although there is no consensus, several authors have shown a decreased REE adjusted for FFM of about 5% in older patients [32,33,34]. This difference could be related to the heterogeneity of FFM, which includes tissues with a higher metabolic rate (heart, liver, kidneys, and brain) and others with a lower metabolic rate as muscle mass. Muscle metabolism may account for only 25% of REE [32]. The decline in REE with aging may be explained by an increased proportion of organ tissue compared to muscle mass as well as a decline in total muscle mass in proportion to FFM [29,30]. However, the relative contribution of these two factors in the decline of REE with age remains to be clarified. It is also important to determine whether the contribution of the REE of organ tissues is higher in acute or subacute situations in order to better understand the energy needs and malnutrition mechanisms of older patients who are often frail and suffer from diseases with various degrees of inflammation. These situations are frequently observed in older hospitalized patients and could be responsible for an increase in energy requirements due to an increase in the energy expenditure of the organ tissue.

Disease burden/inflammation and underlying hypermetabolism with elevated REE has been identified as one of the two etiological criteria for the diagnosis of malnutrition according to a recent international consensus [33]. Acute or subacute medical conditions may increase energy expenditure by inflammation, drugs, fever processes, catecholamines, or cortisol production [33,34,35]. Schrack et al. showed an increase in REE with the number of chronic diseases in a study with “healthy” volunteers from the Baltimore Longitudinal Study of Aging (BLSA) cohort [36]. Although they considered various hypotheses, proinflammatory and catabolic state was put forward to explain the increased REE. However, it was not envisaged that the relationship between REE and inflammatory status would be explored in this study. Inflammation has been found to be associated with an increase in energy expenditure in certain situations, such as cancer [37] or chronic renal failure [38].

Exploratory Spearman’s partial correlations on the 19 patients of group A seem to suggest that higher inflammatory markers on admission (expressed by a lower CRPdiff value given negative sign) might be associated with increased REE, while no association was found with TEE. It could be hypothesized that a reduction in physical activity directly related to the context of hospitalization and possibly associated with increased REE might result in relatively stable TEE. It must be noted, however, that time scales for these measurements were not strictly superimposed given that the DLW method measures the body isotope clearance over several days, while results of REE were obtained instantaneously on the first day of inclusion. Further studies with repeated calorimetry measurements throughout hospitalization along with inflammatory markers would be needed to better study the associations of REE and inflammation. Unfortunately, this was not done for feasibility and acceptability reasons.

In our study, FFM seemed to be the main determinant of energy expenditure. From a cohort of 714 healthy subjects of all ages, Geisler et al. studied age-dependent changes in REE in relation to detailed body composition and found that the variance of REE/FFM was explained for only 2% by CRP levels, even in the absence of any evolving disease responsible for inflammation [30]. Conversely, many reports did not confirm the reality of hypermetabolism and increased REE in hospitalized elderly patients in acute or subacute situations. In a study of hospitalized elderly subjects in acute or rehabilitation care unit, REE was found to be very close to ours [39].

In most published reports that studied REE in patients with chronic diseases in the absence of acute event with only mild to moderate degrees of inflammation, REE was found to be similar to that of healthy elderly people when adjusted for FFM [10,12,40,41]. These results may be explained by the more pronounced decrease in FFM in these patients with inflammation and malnutrition. In underweight, malnourished older patients with BMI below 20 kg/m^2^, hypometabolism was observed, which may be due not only to a decrease in FFM but also to a decline in functional status [42]. This suggests that sedentary lifestyle may also explain the decline in REE in addition to the decrease in FFM, even though physical activity was not measured in their study [42]. In our study, very low PAL may also explain the low REE that we observed.

In a review of 19 studies conducted in 1256 hospitalized patients where sepsis was frequently encountered and in whom REE was measured by indirect calorimetry, no clear relationship could be established between severity of illness and hypermetabolism [43]. Another review considered REE and energy requirements in 2450 healthy, sick, and underweight older patients [9]. The REE measured by indirect calorimetry, after adjusting for body weight and FFM, was similar in healthy and sick older people. Heterogeneity was observed between the different situations. Indeed, the average REE was even lower in the hospitalized population. Likewise, the mean REE per kg FFM was not different between healthy and sick older patients, with 28 ± 3 and 29 ± 2 kcal/kg FFM/d, respectively [9]. These values are also very close to our own results (29 ± 3 and 31 ± 4 kcal/kg FFM/d, respectively).

Some authors consider that REE is increased with frailty and may have prognostic significance. In a study involving old women from Women’s Health and Aging Study (WHAS II) [44], the average REE was 1119 ± 205 kcal/d. A high variability of REE was observed in frail subjects according to Fried’s criteria, suggesting a possible relationship with frailty for both hyper- and hypometabolism situations, which does not allow clear conclusions to be drawn. In the study by Fabbri et al. [45] performed from the BLSA cohort, an increase in REE was predictive of multimorbidity; however, again, the average age of 68.2 years makes the studied populations not really comparable to ours. In two different age groups of 60–74 and 90 years and more from the Louisiana Healthy Aging Study, Kim et al. found an association between REE and a frailty index based on 34 health and functional status variables but only in patients older than 90 years of age [46]. This could suggest an increased energy expenditure in unhealthy very old patients. However, such increase was not observed in the control group of our study, which consisted of frail elderly subjects living in nursing homes. In Kim’s study, TEE was also measured by the doubly labeled water method, and the authors did not find the same results for TEE, with no association between frailty index and TEE [46]. Therefore, it is not possible to make firm conclusions about the increase in energetic needs of this population. Lammes et al. found values of 1174 kcal/d (29 kcal/kg FFM/d) for REE in elderly nursing home patients with multiple diagnoses [47]. These values, like those of the studies previously mentioned, are also very close to our results. Interestingly, they found an energy intake/REE ratio of 1.27, which is very close to the TEE/REE ratio that we have determined. Despite the need to better understand energy needs and their components [48], including PAL in very elderly subjects, few studies that have measured TEE using the DLW method are actually available, with only 248 patients aged 80 or over identified in a recent literature review [19].

Among nonagenarians living at home, the TEE/REE ratio averaged 1.19 for women and 1.36 for men, indicating very low levels of physical activity, especially as there was no decline in the REE relative to a population of septuagenarians [49]. In a longitudinal study, TEE was reassessed nearly seven years later in 83 participants whose mean initial age was about 74 years. A decline of about 100 kcal of energy expenditure related to physical activity was observed each year. The decline during the eighth decade was associated with higher age, poorer physical condition with reduced walking speed, and a greater decrease in muscle mass during follow-up. The occurrence of intercurrent diseases and inflammation were not related to changes in TEE [50]. In another longitudinal study conducted from the Health ABC study cohort on 302 patients aged 70 to 82 years living in a healthy community [51], PAL in only the highest tertile was associated with a reduction in mortality after eight years of follow-up, while neither TEE nor REE was associated with mortality. In patients with hypermetabolism linked with colorectal cancer, TEE was again not increased [52].

Finally, the energy requirements of a population of elderly subjects hospitalized in acute or subacute situations are very much below the dietary reference intakes (DRIs) [53]. If these requirements remain at about 1.7 REE in healthy elderly subjects aged about 75 years living in the community as we or others have shown previously [16,54], they are significantly decreased beyond 80 years of age below the 25th percentile PAL value of 1.49 as established by the Scientific Advisory Committee on Nutrition (SACN 2011) [55], regardless of any acute condition. A more pronounced decline in REE in very old subjects could even be a marker of frailty and sarcopenia as suggested by the results of a recent study [56]. In patients with esophageal cancer, not only was the REE not increased [57], but a low REE was also associated with worse survival at five years [58].

Despite equilibrium of the energy balance with similar values between energy intakes and energy needs as well as the stability of intakes over the two weeks, a significant decrease in weight was observed in our study. This finding suggests that mechanisms other than inflammation alone could be involved and that energy intake must be higher to avoid weight loss. Among these, variations in hydration levels, lack of functional recovery, an increased time of bed rest, the initial pathology, and lower nutritional reserve could be hypothesized. The precision of the measurement methods used is a strength of our study. However, some limitations need to be pointed out. The limited patient numbers due to the high cost of the DLW technique and cross-sectional design are the main limitations of our study. Furthermore, the absence of repeated measures of REE throughout hospitalization does not allow definite conclusions to be drawn on the possible associations between REE and inflammation status in these patients.

## 5. Conclusions

Our results suggest that TEE is not increased in hospitalized older patients with acute condition and inflammation. We have shown that the energy requirements should be at least 1.3 × REE in these patients with nearly 30 kcal/kg FFM for REE. Energy intake here is just sufficient to cover the needs as measured with DLW but possibly not to prevent weight loss due to other incompletely elucidated mechanisms.

## Figures and Tables

**Table 1:** Characteristics, body composition, and nutritional and inflammatory parameters of the groups.

	Group A			Group B			All		
	*n*	Mean	SD	*n*	Mean	SD	*n*	Mean	SD
Age (years)	19	83.6	5.1	55	83.1	5.6	74	83.2	5.4
Weight (kg)	19	63.1	13.2	55	64.0	11.6	74	63.8	12.0
BMI (kg/m²)	19	24.1	3.7	55	27.0	4.3	74	26.3	4.4
Fat-free mass (kg)	19	39.0	7.7	55	37.9	6.2	74	38.1	6.6
Fat-free mass (%)	19	62.3	7.2	55	59.8	6.9	74	60.4	7.0
Fat mass (kg)	19	24.2	8.0	55	26.2	7.9	74	25.6	7.9
Fat mass (%)	19	37.7	7.2	55	40.2	6.9	74	39.6	7.0
Albumin (g/L)	16	29.0	5.0	51	40.9	2.4	67	38.0	6.0
Prealbumin (g/L)	19	0.2	0.1	50	0.3	0.0	69	0.2	0.1
Baseline CRP (mg/L)	19	61.6	32.3	55	0.5	0.0	74	16.2	31.3
CRP at day 14 (mg/L)	19	22.4	27.9	-	-	-	19	22.4	27.9

SD: standard deviation; BMI: body mass index; CRP: C-reactive protein.

**Table 2:** Anthropometric characteristics in men and women.

		Group A			Group B			All		
		*n*	Mean	SD	*n*	Mean	SD	*n*	Mean	SD
Age (years)	All	19	83.6	5.1	55	83.1	5.6	74	83.2	5.4
	Women	13	83.3	4.9	49	83.5	5.5	62	83.5	5.3
	Men	6	84.2	5.8	6	79.8	6.0	12	82.0	6.1
Weight (kg)	All	19	63.1	13.2	55	64.0	11.6	74	63.8	12.0
	Women	13	57.1	8.2	49	62.9	10.9	62	61.7	10.6
	Men	6	76.1	13.0	6	72.8	14.8	12	74.4	13.4
BMI (kg/m²)	All	19	24.1	3.7	55	27.0	4.3	74	26.3	4.4
	Women	13	23.3	3.6	49	27.1	4.3	62	26.3	4.4
	Men	6	26.0	3.6	6	26.6	5.3	12	26.3	4.3
Fat-free mass (kg)	All	19	39.0	7.7	55	37.9	6.2	74	38.1	6.6
	Women	13	34.7	3.3	49	36.5	4.2	62	36.1	4.1
	Men	6	48.2	6.1	6	49.1	8.6	12	48.7	7.2
Fat-free mass (%)	All	19	62.3	7.2	55	59.8	6.9	74	60.4	7.0
	Women	13	61.4	6.9	49	58.8	6.4	62	59.3	6.5
	Men	6	64.2	8.0	6	68.0	5.1	12	66.1	6.7
Fat mass (kg)	All	19	24.2	8.0	55	26.2	7.9	74	25.6	7.9
	Women	13	22.4	7.1	49	26.5	7.9	62	25.6	7.8
	Men	6	27.9	9.2	6	23.6	7.9	12	25.8	8.5
Fat mass (%)	All	19	37.7	7.2	55	40.2	6.9	74	39.6	7.0
	Women	13	38.6	6.9	49	41.2	6.4	62	40.7	6.5
	Men	6	35.8	8.0	6	32.0	5.1	12	33.9	6.7

SD: standard deviation; BMI: body mass index.

**Table 3:** Energy balance.

	Group A			Group B			All		
	*n*	Mean	SD	*n*	Mean	SD	*n*	Mean	SD
Energy intake (kcal/d) ^1^	19	1420	280	0			19	1420	280
REE (kcal/day)	19	1184	215	55	1125	193	74	1140	199
REE (kcal/kg FMM/d)	19	31	4	55	29	3	74	30	3
TEE (kcal/d)	19	1497	209	0			19	1497	209
Physical Activity Level ^2^	19	1.3	0.2	0			19	1.3	0.2

^1^ Energy intake measured during the first three days of hospitalization; ^2^ PAL calculated as TEE/REE. REE: resting energy expenditure (measured by indirect calorimetry); FFM: fat-free mass; TEE: total energy expenditure (measured by doubly labeled water method).

**Table 4:** Associations of fat-free mass and C-reactive protein with resting energy expenditure.

Covariates	Regression Coefficient (95% CI)	*p*-Value
Fat-free mass (per 5 kg increment)	103.74 (69.37; 138.10)	<0.001
C-reactive protein (per 20 unit increment)	20.23 (−2.93; 43.38)	0.086
Age (per five-year increment)	−30.50 (−57.99; −3.00)	0.030
Sex (women vs. men)	16.60 (−100.79; 133.99)	0.779
Resting energy expenditure (men vs. women)	1108.04 (995.42; 1220.67)	<0.001

**Figure A1:**
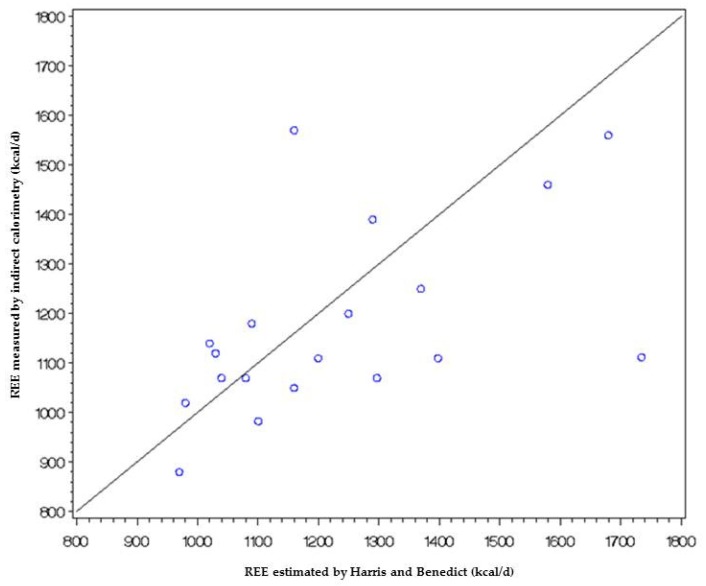
Correlation between indirect calorimetry and Harris and Benedict estimation of REE.

**Figure A2:**
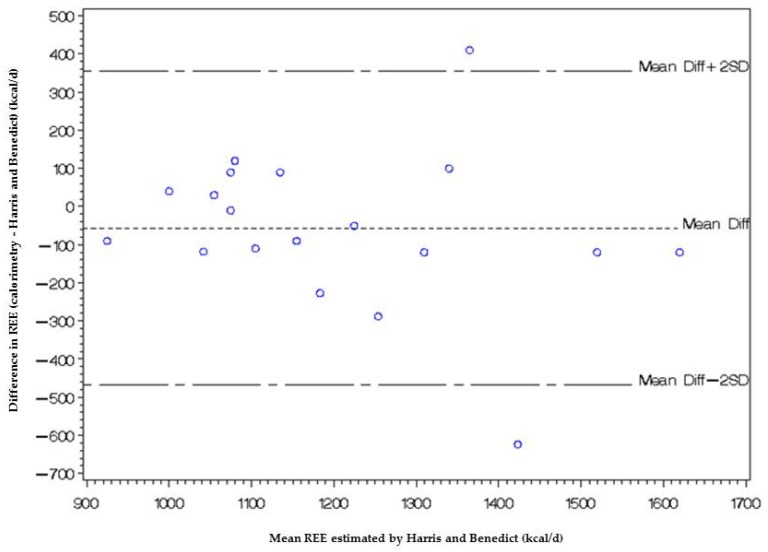
Bland and Altman plot of consistency between REE estimated by Harris and Benedict and indirect calorimetry measurement methods.

**Figure A3:**
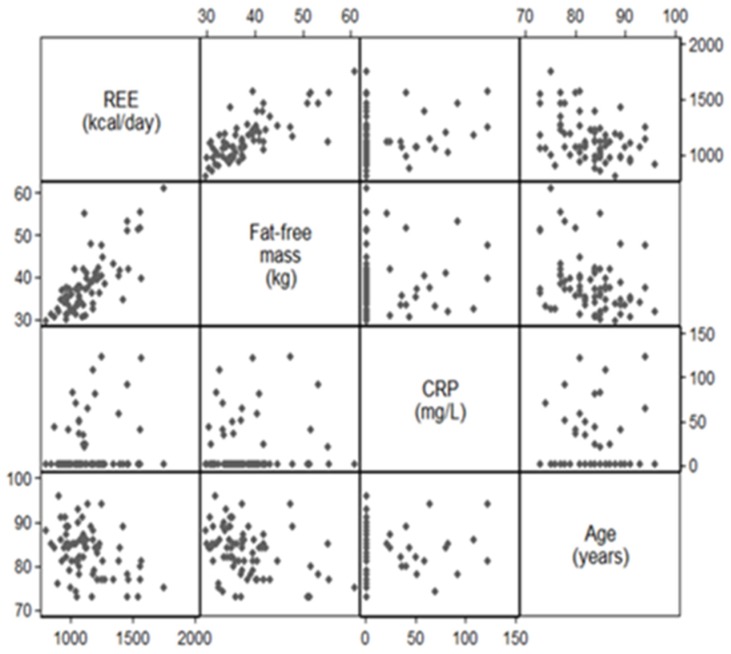
Associations of fat-free mass, CRP and age with resting energy expenditure (REE): results of the bivariate analysis.

## Appendix A

Consistency between calorimetry and Harris and Benedict measurement methods of REE in kcal/day.

The first graph (Figure A1) represents the results of indirect calorimetry versus Harris and Benedict estimation of REE. This graph shows poor agreement between the two methods.

In the Bland-Altman plot below (Figure A2), the difference between the measurements of REE using the two methods (calorimetry-Harris and Benedict method) is represented as a function of the average of the two REE measurements.

This graph shows that there is a relationship between the difference and the mean, with the difference increasing as the mean increases. This implies that there is a link between the measurement error and the true value of REE.

The bias between the two methods is estimated by averaging the difference, −57.16 kcal/day with a standard deviation of 205.45 kcal/day.

Assuming that the differences have a normal distribution, the concordance limits are Mean Diff − 2 × SD = −468.05 kcal/day and Mean Diff + 2 × SD = 353.74 kcal/day.

If these limits are too large for clinical use, the consistency between methods is not good.

## Appendix B

Associations of fat-free mass, CRP and age with resting energy expenditure (REE): results of the bivariate analysis.

This figure pictures the results of the bivariate analyses prior to the modelisation. There was a good correlation between FFM and REE, but correlation was low between CRP levels and REE (Spearman correlation coefficient 0.71 and 0.17, respectively)
